# Properties of Polyethylene Terephthalate (PET) after Thermo-Oxidative Aging

**DOI:** 10.3390/ma14143833

**Published:** 2021-07-08

**Authors:** Robert Panowicz, Marcin Konarzewski, Tomasz Durejko, Mateusz Szala, Magdalena Łazińska, Magdalena Czerwińska, Piotr Prasuła

**Affiliations:** 1 Institute of Mechanics and Computational Engineering, Faculty of Mechanical Engineering, Military University of Technology, 2, Gen. Kaliskiego Str., 00-908 Warsaw, Poland; robert.panowicz@wat.edu.pl; 2 Institute of Materials Science and Engineering, Faculty of Advanced Technologies and Chemistry, Military University of Technology, 2, Gen. Kaliskiego Str., 00-908 Warsaw, Poland; tomasz.durejko@wat.edu.pl (T.D.); mateusz.szala@wat.edu.pl (M.S.); magdalena.lazinska@wat.edu.pl (M.Ł.); 3 Military Institute of Armament Technology, Prym. S. Wyszynskiego 7 Str., 05-220 Zielonka, Poland; czerwinskam@witu.mil.pl (M.C.); prasulap@witu.mil.pl (P.P.)

**Keywords:** aging resistance, PET, thermo-oxidation aging, thermal and mechanical properties

## Abstract

The influence of the thermo-oxidative aging semi-crystalline polyethylene terephthalate process on the thermal and mechanical properties was analysed in the article. For this purpose, PET was aged at 140 °C for 21, 35 and 56 days. The research showed that as a result of aging, the amount of the crystalline phase increases by about 8%, which translates into the properties of the aged material. The glass transition and melt temperature of lamellar crystals formed during first and second crystallisation increase with aging. The mechanical properties of the material were analysed in the temperature range of 25 to 75 °C. The tests were showing an increase in Young’s modulus and a decrease in elongation at the break as a result of aging. This phenomenon was particularly visible during tests at 75 °C and during the morphological observation of the fracture surface, where the fracture character of the material changes from ductile to brittle. In the case of the material aged for the longest time, the temperature has a negligible influence on the elongation at break.

## 1. Introduction

Polyethylene terephthalate (PET) is one of the most popular thermoplastic polymers and is used primarily for the production of clothing fibres, tanks, bottles and also as a construction material. The annual production of this material in 2016 was 50.01 MMT [1] and it is forecast to rise to 87.16 MMT by 2022.

Amorphous PET is used for the production of bottles and packaging due to its high transparency, which is very similar to that of glass. In other cases, a semi-crystalline state of polymer is used, which has a milky white colour and is opaque.

Due to such factors as good mechanical properties (abrasion resistance, dimensional stability-creep resistance, easy processing of details and their surfaces, high impact strength) even at low temperatures (<−70 °C), low water absorption and resistance to inorganic chemicals, PET is widely used in a variety of industries (machine, automotive, electromechanical, electronic). High resistance to various environmental factors along with an absence of harmful low molecular weight substances make it widely used in applications where it comes into contact with food (food industry, food packaging, household appliances).

The change in the properties of PET, as well as that of other polymers, is often influenced by the environment, causing its properties to degrade mainly through random scission of the polymer chains. Depending on the environmental conditions, thermal, thermo-oxidative, chemical, radiative, biochemical and hydrolytic degradation processes can be distinguished. The chemical processes during the thermal degradation of PET were investigated by Holladn and Hay using FTIR [2]. The presence of diethylene glycol and isophthalate units has a significant influence on the process, increasing the chain flexibility and creating more favourable bond angles. The degradation process involves the formation of volatile degradation products. Turnbull, Liggat and MacDonald [3] also dealt with this topic. The conducted research showed that the formation of cyclic oligomers is the main reaction takes place during the thermal degradation process. Pirzadeh, Zadhoush and Haghighat investigated the influence of temperature and humidity on the properties of PET fibres and granules [4]. At temperatures lower than T_g_, they did not observe any material degradation. On the other hand, the course of the hydrolysis process depends on the moisture level, and water is more readily absorbed by the fibres than the granules. Accordingly, the fibres undergo hydrolytic degradation faster, which is not affected by temperature. The influence of chemical degradation on the mechanical properties of the fibres used to reinforce concrete was investigated by Machovie et al. [5] Ciera et al. investigated the influence of incorporated spores on the mechanical and morphological properties of PET fibres [6].

In addition to changes in the properties of PET caused by degradation processes, this material is also susceptible to physical aging below the glass transition temperature caused by the slow drive of quenched material at a thermodynamically non-equilibrium state to equilibrium. This is related to the relaxation processes with characteristic, different time constants [7,8]. It results in a reduction in entropy, enthalpy and specific volume with an increase in yield stress and tensile and flexural module. Hay investigated the effect of the crystalline phase on the behaviour and properties of PET. It turned out that the crystalline phase limits mobility of the chain segments, influencing the macroscopic properties of the material [8]. Zhao et al. used the physical aging process to analyse the amorphous phase in semi-crystalline polyethylene terephthalate [9]. The research shows that two types of areas filled with the amorphous PET phase can be distinguished. The first area—the free amorphous region—can evolve during aging and is mainly responsible for changes in properties, transforming into the second area, the constrained amorphous region. The total number of the amorphous phase remains practically constant. Sato and Sprengel studied the dynamics of the relaxation process below the glass transition temperature and determined the constants of individual relaxation processes [7]. Farhoodi et al. investigated the physical aging process at 25 °C and 45 °C. No changes were observed at lower temperatures, while the higher temperature allowed for the observation of the relaxation processes in the material [10].

Tests using the Differential Scanning Calorimetry technique show that depending on the crystallization, the recrystallization process of PET and the subsequent annealing (isothermal crystallization), endothermic peaks can appear on thermograms [11,12,13]. This phenomenon is characteristic not only for PET, but also for other polymers [14,15]. In most cases, two endothermic peaks are observed. The first endothermic peak is associated with secondary crystallization, while the second peak is associated with the melting of crystallites formed during the primary, first crystallization [11,13]. The lamellar crystals formed during primary crystallization are characterized not only by greater thickness, but also by a structure closer to an ideal crystal. In contrast, structures formed at lower temperatures are thinner, with more structural defects and lower thermal stability [11,16,17].

Tang et al. showed that lamella growth is the dominant process for annealing in the temperature range of 180 to 220 °C [18]. Annealing at higher temperatures increases the thickness of the lamellae formed during primary crystallization. According to Ziyu and Hay, diffusion processes are responsible for the increase in lamella thickness when annealing above the crystallization temperature and below the melting temperature [19].

The kinetics of the isothermal crystallization process was studied by Zheng et al. [20] and by Zendehzaban and Shamsipur [21]. Research has shown that increasing the aging temperature significantly affects the quality of crystallites as well as the melting point. Additionally, materials with shorter chains crystallize more easily.

Due to the very high production of plastics and the resulting increase in environmental pollution, the possibilities of their processing and reuse were addressed. This is connected not only with the processing of the material, but also with the determination of the influence of the method of processing the material on its properties. The relatively simple way of recycling PET meant that it is reprocessed in large amounts, and numerous works related to recycled PET can be found [22,23,24,25].

The works conducted by the researchers are also aimed at the modification of PET with additives improving its properties [26,27,28,29] or enabling acceleration of the degradation process [30,31,32].

Thermo-oxidative aging is the main, basic process that causes the destruction of structural elements made of PET. For this reason, the aim of the presented article is to show the influence of the thermo-oxidative aging process of the polyethylene terephthalate on its properties in the temperature range at which this material is commonly used. Typical commercially available PET was used in this study after polycondensation. Intrinsic viscosity (IV) and the average molecular weights of such a material are 0.60 dL g^−1^ and 24.8 kg mol^−1^, respectively [13,33,34].

## 2. Experimental Part

### 2.1. Materials Preparation

The PET sheet was cut into pieces, which were then placed in an oven heated to a temperature of 140 °C in such a way that air could freely flow around each of the pieces. The degradation process of PET at temperatures of 110 and 120 °C is relatively slow; therefore, a higher temperature was adopted [35]. The pieces were removed from the oven after 21, 35 and 56 days. Then, test specimens were made from unaged and aged PET.

### 2.2. Differential Scanning Calorimetry

The TA Instruments DSC 250 apparatus (New Castle, DE, USA) was used to perform the differential scanning calorimetry (DSC) tests. Measurements were carried out in an inert gas atmosphere at a heating rate of 10 °C/min, in the range of 50 to 300 °C.

### 2.3. Fourier-Transform Infrared

Fourier-transform infrared (FTIR) spectra were collected by a Thermo Nicolet iS5 FTIR Spectrometer (Waltham, MA, USA), equipped with a single-reflection accessory (iD7) for attenuated total reflection (ATR) measurements, with diamond crystal. Thirty-two accumulated spectra were recorded for each specimen at a resolution of 4 cm^−1^ from 4000 to 600 cm^−1^ of wavenumber. IR spectra were analyzed with the help of OMNIC software (version 9.11.745) from Thermo Scientific (Waltham, MA, USA). At least 32 measurements per material were performed at different locations of the plate, in order to obtain representative results. The obtained spectra were used to determine Carbonyl Index (CI). The CI values were calculated from the ratio between the integrated band absorbance of the carbonyl group (C=O, 1712 cm^−1^) and the methylene group (CH_2_, 1408 cm^−1^) (1) [36]. The area under the band is calculated through the Omnic software using the peak analysis tool. This index allows the characterization of the degradation process in polymeric materials based on the baseline method.

(1) $$\mathrm{CI}=\frac{\left(\mathrm{Absorbance}\;\mathrm{at}\;1712\;{\mathrm{cm}}^{-1}\;\right)}{\left(\mathrm{Absorbance}\;\mathrm{at}\;1408\;{\mathrm{cm}}^{-1}\right)}$$

### 2.4. Dynamic Mechanical Analysis

The experiment of dynamic mechanical analysis (DMA) of cuboid samples was conducted using TA Instruments DMA Q800 analyser (New Castle, DE, USA). The dual cantilever clamps were used to test the mechanical properties polymer. The measurements were carried out within the temperature range from −70 °C to 255 °C, at a heating rate of 3 °C/min and with frequency of 1 Hz. The amplitude of test sample deformation was established at 10 µm. The tests used cuboidal specimens, cut from pieces of PET sheets, with the following dimensions: 60 mm, width of about 10 mm, thickness of about 3 mm.

### 2.5. Mechanical Analysis

The static tensile test was carried out on the Instron 8862 electromechanical universal test system (Instron, Norwood, MA, USA). The maximum displacement range of this system is ±100 mm and the maximum piston speed is 300 mm/min. In order to perform the test in a variety of positive temperatures, the Instron temperature chamber, installed on the test system, was used. The chamber allows conducting the test at temperatures from −100 °C to 320 °C.

The test system automatically recorded the loading force and the position of the traverse in time with 50 Hz frequency. The tests were performed by applying displacement-controlled loading. The speed of the traverse was equal to 10 mm/min and a strain rate during the test was constant and equal to 7 × 10^−3^ 1/s. The forces were recorded by the test system, while the displacements were registered with use of the Instron extensometer.

The dimensions of the samples were determined on the basis of the ISO 527-2:2012 standard concerning a determination of the tensile properties of moulding and extrusion plastics. The 1BA test specimen was used, because of the limited space of the temperature chamber. The water jet cutting technique was utilised to prepare the test samples.

The tensile tests were carried out on the basis of the PN-ISO 527:2007 standard. According to the standard, the tests were performed with the use of the dog bone samples at a constant strain rate. Tensile stress was calculated as the force related to the initial cross section area of the gauge length of the tested sample. Tensile strength is defined as the maximum recorder tensile stress and elongation at break as the gauge length deformation at break. The effect of transverse deformations of the sample during the test is not taken into account.

The samples were tested in the following temperature range: 25 °C, 50 °C and 75 °C. After mounting the samples in the clamps of the testing system and closing the climate chamber, about 15 min were allowed to settle and normalize the temperature inside the chamber. Measurements of the temperature inside the chamber were performed with the use of the two K-type thermocouples: one placed in the working field of the chamber and the other fixed between two layers of the tested material (two pieces of the test material were applied to each other and a thermocouple was placed between them). The tests were carried out when the control system was showing a constant, set temperature. According to the chamber specification, it allows temperature stabilization in the range of ±2 °C, and the permissible K-type thermocouple error is ±1.5 °C.

### 2.6. Scanning Electron Microscopy

A model FEI Quanta 3D FEG scanning electron microscope (Hillsboro, OR, USA) was used to analyse the surface fracture of PET samples. The analysis of the failure mechanisms was carried out by a direct observation of the topography of fracture surfaces of each PET variant, i.e., as-received, aged during 21, 35, and 56 days and then mechanically tested at 25 °C, 50 °C and 70 °C. The observations were conducted at magnification 200× and 500× in a low-vacuum mode.

### 2.7. Light Microscope

The macro-observations of the fracture PET samples after tensile tests were performed on a Keyence VHX-950F light microscope (Osaka, Japan) with a magnification of 20×.

## 3. Results

Density measurements by aerometric method showed a slight increase in density with material aging (Table 1).

**Figure 1:**
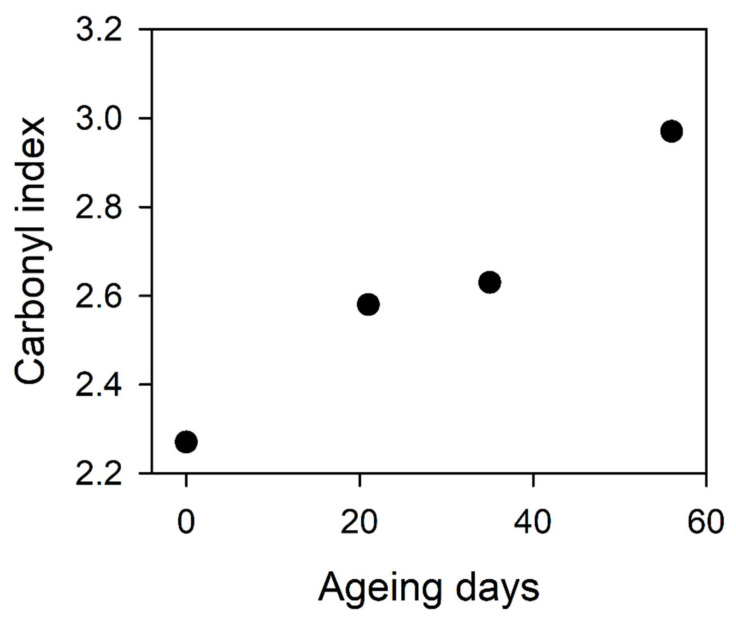
Carbonyl index as a function of ageing time for a series of PET samples.

The CI as a function of the ageing time plot is shown in Figure 1. The trend in the CI changes associated with the degradation process is clear and visible. The starting value of the CI indicates that the non-aged polymer is partially oxidated, e.g., after high-temperature processing like extrusion or thermoforming.

**Figure 2:**
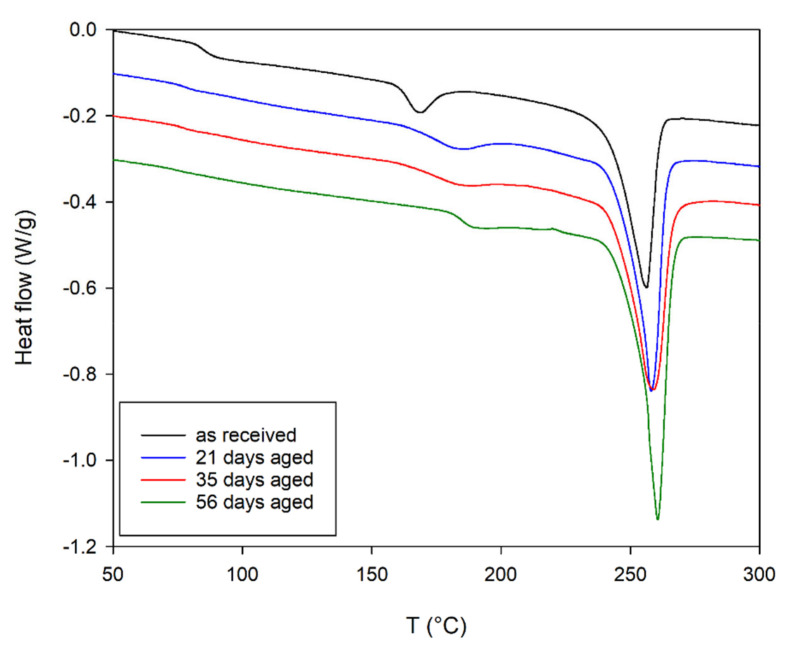
DSC thermogram of as-received and unaged PET, curves have been shifted in relation to each other in order to better visualize the results.

DSC thermogram of aged and unaged PET is presented in Figure 2, while the parameters determined from them are included in Table 2. The midpoint glass transition temperature (*T_g_*) of the unaged PET is equal to 85.4 °C (Table 2). This temperature decreases to 76.1 °C for aged materials. The change in enthalpy associated with the glass transition process also decreases.

The first endothermic peak temperature (*T_I_*) increases from 168.4 to 185.1 °C for PET as-received and 56 days aged, respectively. On the other hand, the change in enthalpy of this process (Δ*H_I_*) initially increases slightly to later decrease to 3.35 J/g with a continuous widening of the peak.

The softening point of the second endothermic peak (*T_m_*) and the change in enthalpy (Δ*H_m_*) associated with this process increase with the aging of the material from 254.9 °C, 31.4 J/g to 260.6 °C and 42.8 J/g for PET specimens as-received and aged for 56 days, respectively. This is significantly less than the temperature change of the first endothermic peak. A little difference in the sample aged for 35 days can be observed. In this case there is no increase in Δ*H_m_*, with a simultaneous slight widening of the peak.

The amount of crystallinity in the sample can be determined from the relationship:

(2) $$\chi_{c}=\frac{\Delta H_{i}}{\Delta H^{0}}\cdot 100\%$$

where: $\Delta H^{0}$ is the heat of the melting of a sample consisting only of the crystalline phase, $\Delta H_{i}^{}$ is the heat of melting first (*i* = *I*) or second (*i* = *m*) endothermic peak.

The values of the heat of melting of a purely crystalline sample reported in the literature differ quite significantly, affecting the determination of the degree of crystallinity in the sample. The values vary from 117.7 to 140 J/g [5,22,26,32,35]. The heat of melting of a purely crystalline sample value of 140 J/g was used for further calculations. The calculated degree of crystallinity formed during primary (**χ_cm_**) and secondary (**χ_cI_**) crystallization on the basis of the relationship (2) are included in Table 3. During the first phase of the aging process (up to 21 days), the growth of crystallites can be observed (previously formed during primary and secondary crystallization). At the same time, the structure of the crystallites formed during the secondary crystallization is significantly improved, which is demonstrated by an increase in the melting point by almost 17 °C. Further aging causes a slight decrease in the number of the poorer quality crystallites from the secondary crystallization, with an increase in the amount of the first crystallization crystallites. Two reasons for this phenomenon can be distinguished, which are partially dependent on each other. The first one concerns the lower temperature stability of this type of crystallite and the second one occurs from a degradation process expressed by index CI (Figure 1).

**Figure 3:**
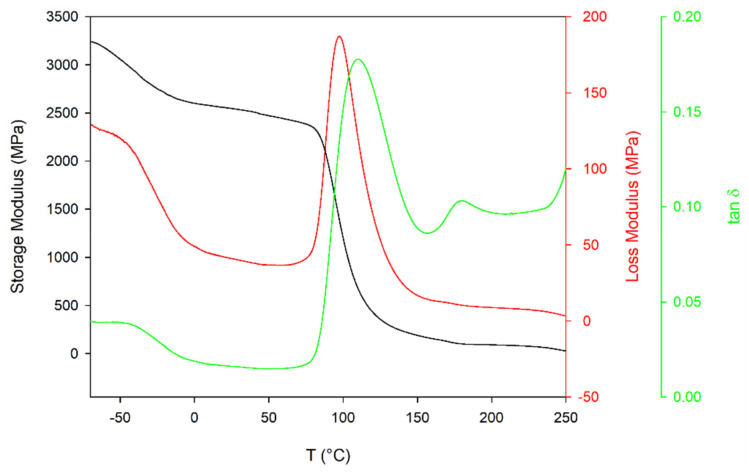
DMA thermogram of as-received PET sample (storage modulus, loss modulus and tan δ vs. temperature).

The dynamic storage (E′), loss modulus (E″) and Tan Delta (tan δ) curves as-received PET were presented in Figure 3. Below 0 °C degrees, the β-relaxation process begins, which has the maximum effect on the parameters E’, E” and tan δ at around −70 °C causing an increase in these parameters from 2600.8 MPa, 48.9 MPa and 0.02 at 0 °C to 3242.3 MPa, 129.3 MPa and 0.04 at −70 °C, respectively. This change is attributed to torsional vibrations of the main chain correlated over a length corresponding to one monomer only at the amorphous phase [37,38,39]. However, it has been shown in [40] that a more complex process can occur in which movements of carbonyl groups (<−70 °C) and phenyl rings (<0 °C) play a role. As the temperature increases, there is α-relaxation associated with the typical glass transition process. During this process, the loss modulus curves reached a maximum at about 187.2 MPa at temperature 97.3 °C, while the maximum tan δ value is reached at a temperature 12.6 °C higher. Above this temperature, on the tan δ curve, there is a second maximum (T_I_ = 179.6 °C) associated with the melting of the crystallites formed during the second crystallization. A further increase in temperature causes a decrease in both the storage and the loss modulus. However, the storage modulus falls more strongly, which causes Tan Delta to increase significantly near the melting point.

**Figure 4:**
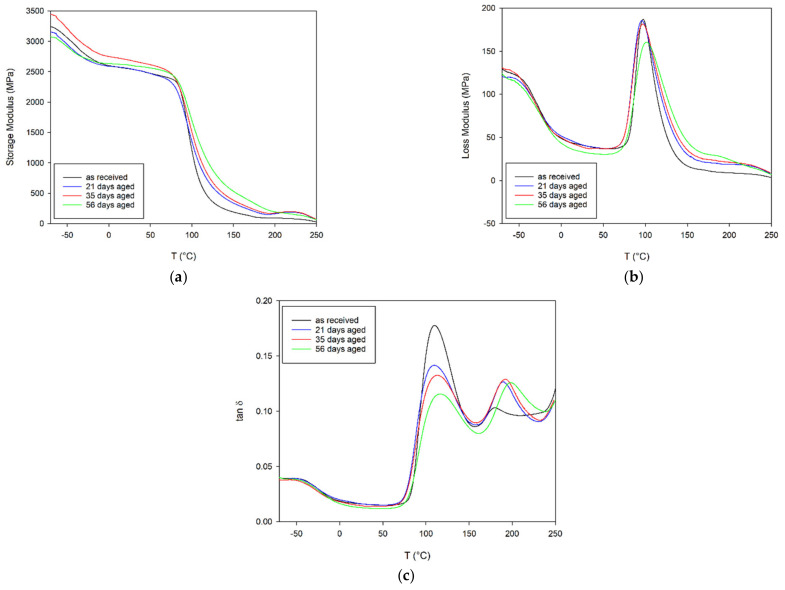
DMA thermogram of unaged and after ageing PET sample; (**a**) storage modulus, (**b**) loss modulus and (**c**) tan δ vs. temperature.

Figure 4 shows the storage modulus, loss modulus and Tan Delta dependences on temperature and ageing, respectively. The storage modulus behaviour in Figure 4a shows that aged PET samples possessed a slightly higher storage modulus compared to unaged PET above the glass transition temperature (T_g_). A sharp decrease in the modules was observed corresponding to the glass transition at around 90 °C. The behaviour of the loss modulus with temperature for the PET specimen is presented in Figure 4b. PET after accelerated ageing had a lower maxima of peak at loss modulus compared to unaged PET. The Tan Delta behaviour is shown in Figure 4c. At the glass transition temperature point, Tan Delta values decreased with the aging of PET samples. Above T_g_, a second smaller maximum associated with cold crystallisation is visible. The value of this maximum is significantly higher for the aged material and is shifted towards higher temperatures.

The glass transition temperature (T_g_) estimated based on the temperature maxima in the loss modulus, and Tan Delta cold crystallisation temperature are presented in Table 4.

**Figure 5:**
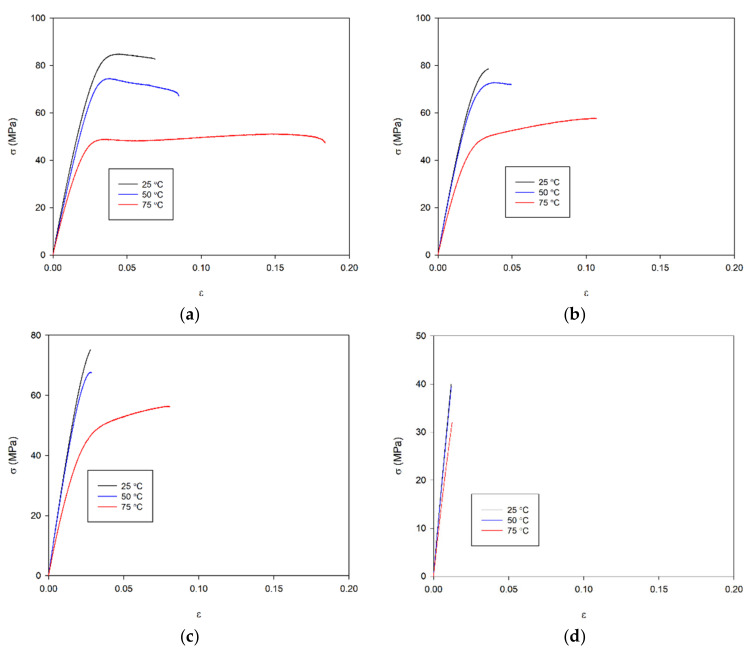
(**a**) Stress-strain curves for different temperatures of as-received PET; (**b**) stress-strain curves for different temperatures of 21 days aged PET; (**c**) stress-strain curves for different temperatures of 35 days aged PET; (**d**) stress-strain curves for different temperatures of 56 days aged PET.

**Figure 6:**
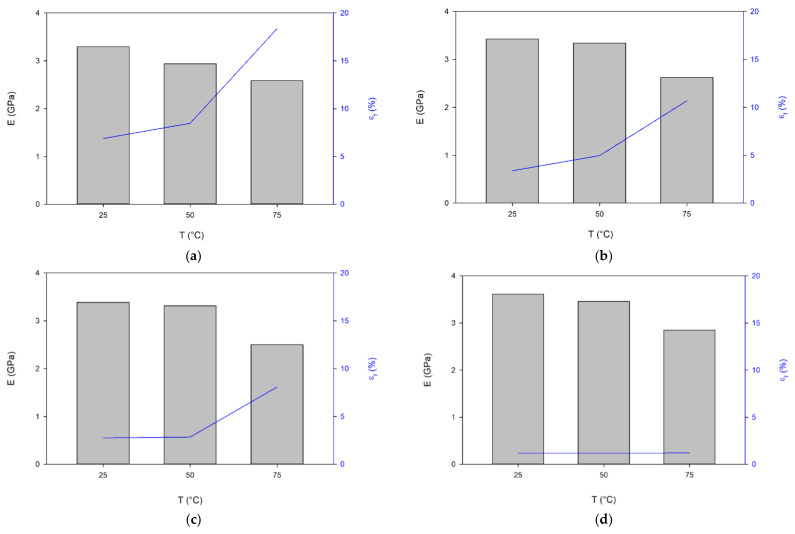
Young modulus (bars) and strain fracture (line) (**a**) as received; (**b**) 21 days aged; (**c**) 35 days aged; (**d**) 56 days aged.

Aging of PET causes an increase in the content of the crystalline phase in the material, an increase in T_g_, T_I_ and T_m_ (Table 2 and Table 4), which translates into the mechanical properties of the material (Figure 5 and Figure 6, Table 5). This process is particularly evident in the stress-strain curves obtained during the tensile test using the universal testing machine Instron 8862 and the climate chamber at higher temperatures of 50 and 75 °C (Figure 5). From a ductile material that breaks at a deformation greater than 18% and a stress of 47 MPa, the material becomes a very brittle material due to aging and breaks at a deformation of about 1.2% and a stress 32 MPa at a temperature of 75 °C (Table 5 and Figure 6). This phenomenon also occurs at lower temperatures, but is less visible.

The increase in the amount of the crystalline phase and the crosslinking between monomers causes a decrease in the mobility of the monomers in relation to each other and a significant reduction in the mobility of individual groups in the monomers, causing an increase in the stiffness of the sample manifested by an increase in Young’s modulus and a decrease in the elongation at break of the specimen. After 21 days of aging, the values of Young’s modulus at 25 and 50 °C came closer to each other (Figure 6). There has been a marked increase in E at the highest temperature of 75 °C. However, this does not exceed 3 GPa. In the case of the longest aged PET (56 days), not only is the material brittle, but the temperature has practically no effect on the elongation at break in the specimen. In contrast, yield stress (σ_Y_) and tensile stress at the break (σ_b_) of the specimen decrease with both an increasing temperature and the aging process (Table 5).

**Figure 7:**
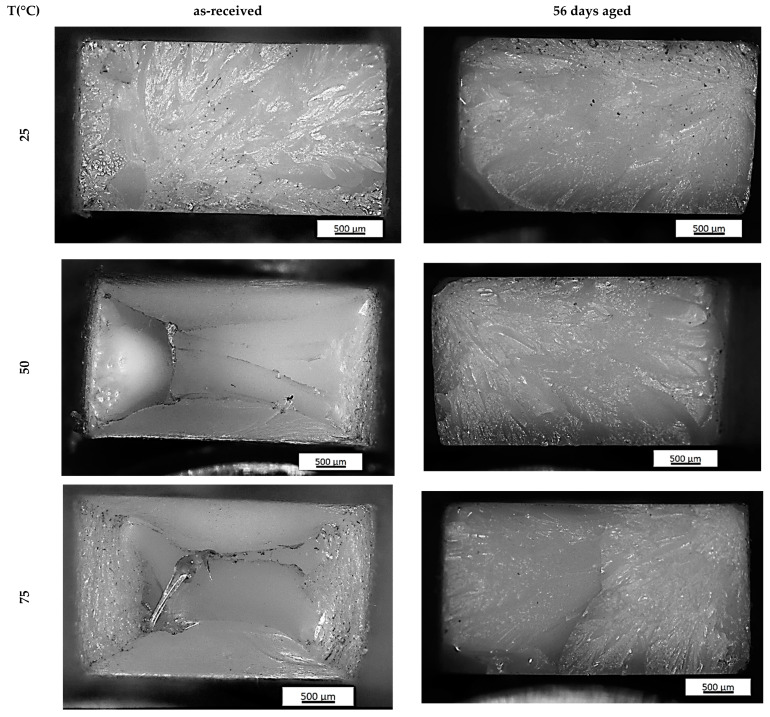
Photo of as-received PET and 56 days aged PET specimen fracture surface tested at different temperatures.

**Figure 8:**
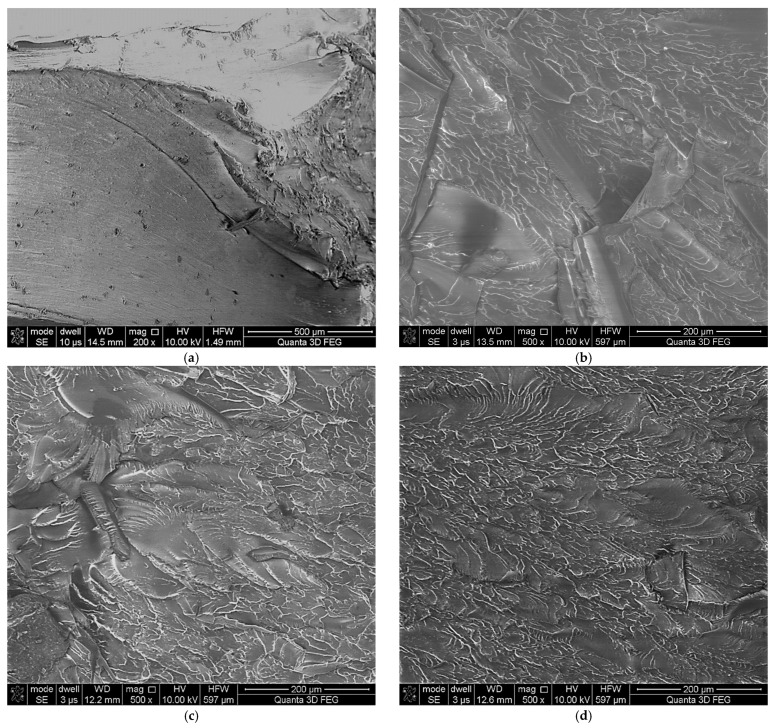
SEM micrographs of specimen fracture surface of as-received PET and 56 days aged PET. The tension test was carried out at 75 °C. Aging time; (**a**) as-received; (**b**) 21 days; (**c**) 35 days; (**d**) 56 days.

The type of fracture slightly changed with aging time for the PET samples tested at room temperature. For the as-received PET sample, the smooth area (known as the mirror zone) characterized by a slow, subcritical crack growth can be observed (Figure 7). The mirror zone directly transforms into hackle regions with a rough surface and are clearly visible in Figure 8 as outward divergent lines pointing along the crack propagation direction. The roughness of the observed hackle regions is the effect of both the occurrence of plastic flow on the fracture surface and the presence of non-coplanar crack surfaces. A similar fracture as for as-received PET samples was noticed for samples subjected to aging during 21 and 35 days and mechanically tested at room temperature. Whereas the clearly different structure was observed for 56 days aging PET (Figure 8). There are only hackle regions with a lot of voids. The plasticity of this variant strongly decreases and is near the zero versus about 4% for as-received PET. Therefore, there was definitely a greater impact on the type of PET sample fracture observed when the tensile temperature was increased. The fracture of the as-received PET sample tested at 50 °C and 75 °C was characteristic of plastic flow (Figure 7). This can be clearly seen in Figure 8 as a smooth textured region along the strain direction. Moreover, some voids are visible. The aging time of 21 and 35 days strongly influences the fracture topography. The conducted light microscope observations reveal the mirror zone and hackle regions for both aging time variants mechanically tested at 50 °C as well as at 75 °C. Furthermore, SEM observations reveal the complex structure of fractures (Figure 8). They consist of the hackle regions with a planar surface, smooth textured areas and parabolic markings. The fracture structure for 56 days of aging PET samples are independent on tensile temperature. When both variants are visible, only the hackle regions correspond with the high brittleness showed in Figure 8b.

## 4. Discussion

Semi-crystalline polyethylene terephthalate has a complex structure. PET consists of lamellar crystals embedded in an amorphous matrix. Some of the chains pass through the crystals (tie-molecules), binding them together [41,42], whereas lamellar crystals are arranged in stacks form higher order structures. The behaviour of a material depends on the internal structure, on both the crystalline and amorphous parts as well as on the structure of the connections between these parts [43]. In turn, the deformation of the polymer is always associated with a change in the microstructure of the material. The polymer complex structure during tension or compression of a sample gives rise to several phenomena, including the shear of lamellar crystals binding together (up to tie-molecule breaking), lamellar crystal rotation, interlamellar separation and homogeneous or inhomogeneous deformation of spherulites [44]. The complex structure results in a state of constant strain rather than constant stress in the loaded material. Strobl and co-workers distinguished 4 characteristic points linking the macroscopic response of a tensile specimen to the phenomena occurring in it [45,46,47]. In the range of the small strain up to the elastic limit (ε = 0.02–0.04) was noticed the onset of isolated inter- and intralamellar slip processes. Next, a change into a collective activity of slips occurs up to ε = 0.1–0.2, which lies slightly above the yield point. For larger deformations, fragmentation of lamellar crystals and the beginning of fibril formation and chain disentanglement was observed.

Young’s modulus and yield stress depend on the level of crystalline phase content in the sample and increase with its content [47]; the skeleton formed by the crystalline part plays a major role in this respect [48,49]. This was also partially confirmed in the presented studies. In the first period of the aging process (21 days), the content of the crystalline phase increased by 4.4% and later also by about 3%. An increase in T_g_, T_m_ and T_I_, density indicates an increase in the crystalline phase and slight scissors of the molecule chains. This means that as a result of the aging process, the crystallite blocks are consolidated and the skeleton stiffness increases. The increase in the stiffness of the skeleton was achieved by an increase in the order inside the lamellar crystals and an increase in the energy of the weakest interactions to the level in which the increase in temperature to 50 °C did not affect these bonds.

At ambient temperature, Young’s modulus changed relatively little, while at 50 °C it increased in the first period by 0.4 GPa, and later these changes have been much smaller (about 0.12 GPa). However, these bonds are so weak that they do not significantly change E at 75 °C, while yield stress and tensile stress at break decrease with both the increasing temperature and the time of the aging process, which is in contradiction to the results presented in [47] and results from the polymer degradation process.

The values of maximum strains suggest that only at 75 °C can fragmentation of lamellar crystals and the fibril formation can occur, but only for as-received and 21 days-aged materials. In other cases, only the collective activity of slips or the onset of isolated inter- and intralamellar slip processes may occur. The increase in ordering in the lamellar crystals, as well as their growth, causes the lamellar crystals not to fragment. As Darras and Séguéla have shown, it is not so much the size of the lamellae that matters as their thickness [50] and the degree of ordering. Moreover, in the process of stretching, tie-molecules embedded in the lamellar crystals are responsible for stress concentration in the crystals in which they are embedded [43]. This can be explained by the fact that tie-molecules embedded in the lamellar crystals create a complex stress field around their fixing. This field can interact with a field from another tie-molecule accelerating the fragmentation process. An increase in lamellar crystal thickness causes an increase in the average distance between the anchor points of tie-molecules on opposite sides of the crystals, and thus the interaction of the fields decreases. Besides, with aging, the ordering in the lamellar crystal increases its strength. In such a situation, the process of fracture of tie-molecules near their anchor point in the lamellar crystal and slip processes is responsible for specimen breakage. From the presented tests, it can be seen that the collective activity of slips depends on temperature, while the onset of isolated inter- and intralamellar slip processes does not depend on temperature (Figure 6).

Finally, it should be noted that the complex structure of PET causes all of the discussed phenomena to occur in the material during experimental tests, but with different intensities. Therefore, the photos of the PET specimen fracture surface (Figure 7 and Figure 8) show both areas associated with ductile and brittle cracking.

## 5. Summary

The influence of the thermo-oxidative aging semi-crystalline polyethylene terephthalate process on the thermal and mechanical properties was analysed. The research shows that as a result of aging, the amount of the crystalline phase increases by about 8%, which translates into the properties of the aged material. The glass transition and melt temperature of lamellar crystals formed during first and second crystallisation increase with aging. The tests were showing an increase in Young’s modulus and a decrease in elongation at break as a result of aging in the temperature range from 25 to 75 °C. This phenomenon was particularly visible during tests at 75 °C and during the morphological observation of the fracture surface, where the fracture character of the material changes from ductile to brittle. In the case of the material aged for the longest time, the temperature has a negligible influence on the elongation at break. This is due to the increase in the thickness of the lamellar crystals as well as the increase in its degree of ordering. The presented results were justified by an increase in the strength of the skeleton formed by the crystalline and the degree of homogeneity of the lamellar crystals, especially those formed during the second crystallization, as well as its thickness.

## Figures and Tables

**Table 1:** PET density measured by the hydrostatic method.

Parameter	As-Received	21 Days	35 Days	56 Days
ρ (g/cm^3^)	1.387	1.392	1.396	1.402

**Table 2:** PET parameters obtained using the DSC method.

Aging Time	*T_g_* (°C)	*T_I_* (°C)	Δ*H_I_* (J/g)	*T_m onset_* (°C)	*T_m_* (°C)	*T_m end_* (°C)	Δ*H_m_* (J/g)
as-received	85.4	168.9	4.2	242.8	254.9	261.7	31.4
21 days	77.5	185.8	4.3	249.8	258.0	268.8	37.4
35 days	76.3	188.7	3.6	249.9	257.6	265.8	37.3
56 days	76.1	194.5	3.35	252.5	260.7	266.3	42.8

**Table 3:** Degree of crystallinity formed during primary (**χ**_cm_) and secondary (**χ**_cI_) crystallization.

Aging Time	χ_cI_ (%)	χ_cm_ (%)
as-received	3.0	22.4
21 days	3.1	26.7
35 days	2.6	26.6
56 days	2.4	30.6

**Table 4:** PET parameters obtained using the DMA method.

Aging Time	T_g (loss modulus)_ (°C)	T_g (tan δ)_ (°C)	T_I (tan δ)_ (°C)
as-received	96.3	109.2	179.5
21 days	96.2	110.9	189.7
35 days	97.0	113.5	192.3
56 days	100.9	115.7	197.9

**Table 5:** Yield stress (σ_Y_) and tensile stress at break (σ_b_); dark grey background colour—yield stress values determined as for metals; light grey background colour—similar or the same yield and tensile stress values resulting from the change of material properties and its brittle fracture.

Temperature	As-Received		21 Days Aged		35 Days Aged		56 Days Aged	
T (°C)	σ_Y_ (MPa)	σ_b_ (MPa)	σ_Y_ (MPa)	σ_b_ (MPa)	σ_Y_ (MPa)	σ_b_ (MPa)	σ_Y_ (MPa)	σ_b_ (MPa)
25	84.8	82.7	78.6	78.4	75.2	75.2	40	40
50	74.5	67.2	72.8	72	67.7	67.5	39.6	39.6
75	51	47.3	39.1	57.8	37.1	56.3	32	32

## Data Availability

Data sharing is not applicable to this article.
